# Response to the Letter to the Editor Regarding “Assessment of Local Adverse Reactions to Subcutaneous Immunoglobulin (SCIG) in Clinical Trials”

**DOI:** 10.1007/s10875-017-0438-y

**Published:** 2017-09-05

**Authors:** Daniel Suez, Mark Stein, Sudhir Gupta, Iftikhar Hussain, Isaac Melamed, Kenneth Paris, Amy Darter, Christelle Bourgeois, Sandor Fritsch, Heinz Leibl, Barbara McCoy, David Gelmont, Leman Yel

**Affiliations:** 1 Allergy, Asthma and Immunology Clinic PA, Irving, TX USA; 2 Allergy Associates of the Palm Beaches, North Palm Beach, FL USA; 3 Division of Basic and Clinical Immunology, University of California at Irvine, Irvine, CA USA; 4 Vital Prospects Clinical Research Institute, Tulsa, OK USA; 5 IMMUNOe International Research Centers, Centennial, CO USA; 6 LSU Health Sciences Center, Children’s Hospital, New Orleans, LA USA; 7 Oklahoma Institute of Allergy and Asthma Clinical Research, LLC, Oklahoma City, OK USA; 8 Shire, Vienna, Austria; 9 Shire, Cambridge, MA USA; 10 Shire, Westlake Village, CA USA

To the Editor:

In the recent letter to the editor, “Assessment of Local Adverse Reactions to Subcutaneous Immunoglobulin (SCIG) in Clinical Trials” [1], Ballow et al. discuss the inappropriateness of making comparisons of adverse event (AE) and tolerability data from different clinical trials of subcutaneous immunoglobulins (IGs) unless the products are studied contemporaneously within the same study using the same methodology, the same investigators, and the same patient populations. The authors conclude that “given the current difficulties in standardizing methodologies across sites and studies, comparisons of tolerability of different products in reported clinical trials should be avoided.” Their letter focuses on the recent phase 3 clinical trial publication of IG20Gly (Cuvitru®, SCIG 20%, Baxalta US Inc., Westlake Village, CA, USA) by Suez et al., which reports the infusion administration parameters and rates of AEs, and discusses the results within the framework of reported data for other available subcutaneous IG products [2].

We welcome the recommendation to standardize the collection of study data to allow for more straightforward data comparisons. However, our comparative discussion made no assertion of superiority and instead stated that IG20Gly treatment was well tolerated, despite the higher infusion rates and volumes per site than those previously reported with other subcutaneous IG products [2]. The discussion of manufacturing processes, excipients, and infusion supplies in the Suez et al. publication was in reference to reasons the high infusion rates and volumes up to 60 mL/h/site and 60 mL/site, respectively, were achievable with IG20Gly.

In addition, although we agree that drawing conclusions regarding the superiority of a product from direct comparisons of data acquired in trials with different study designs, populations, and methodology is not appropriate, we maintain that it is appropriate to make comparisons of data among trials, with acknowledgment of the limitations, in order to provide reference points to contextualize the results. Indeed, the discussion section of the Suez et al. publication includes a disclaimer, “differences in study design and product concentration may limit direct comparison” [2]. The Borte et al. publication, which reported similar data for IG20Gly in a European patient population, also included such a statement [3].

It is a common approach to reference and compare results from other studies in the discussion in order to provide a reference point. For example, in the publication of the phase 3 data of IgPro10 (Privigen®, a 10% intravenous IG preparation), Stein et al. [4] mention in the discussion section that “the proportion of infusions with reports of temporally associated AEs (21%) in this study compares favorably with data obtained with two other recently studied liquid IVIG preparations: 24.9% (Gammagard Liquid® 10%) and 29.1% (Flebogamma® 5%).” In addition, the publication reporting the phase 3 results for a 20% subcutaneous IG product (Hizentra®) [5] compares the main efficacy results and the annual rate of serious bacterial infections (SBIs) to the rate obtained with the 16% subcutaneous IG product (Vivaglobin®) as well as intravenous IG products Gammagard Liquid® and Privigen®. Hagan et al. also mention the rates reported for a novel IVIG (Gamunex™ 10%) despite the use of different definitions of serious infections in the trials [5]. Furthermore, Hagan et al. state that “in general, the annual rate of SBIs in this study is slightly better than the overall mean of 0.068 SBIs per patient per year observed in the licensing trials of all IVIG preparations approved in the United States since 2000” [5].

The rates of AEs reported by Suez et al. [2] for IG20Gly, in many instances, were considerably lower than those reported by Hagan et al. [5] despite vigorous AE assessment throughout the study. The data collection, definitions, and reporting used in the IG20Gly clinical trials were robust and based on pharmacovigilance guidelines to comply with regulatory definitions [6–8]. Nonetheless, the authors do not make any judgment or draw a conclusion that the much lower AE rates for IG20Gly indicate the superiority of IG20Gly over another IG product. Suez et al. state [2], “the rate per infusion of local AEs deemed related to IGSC 20% (0.015 event/infusion) was much lower than the rates reported with a licensed equivalent IGSC 20% preparation in studies conducted in the USA (0.592 and 0.600 event/infusion, respectively) and in Japan (0.274 event/infusion) and lower than the rates observed in an EU study (0.060 event/infusion).”

In conclusion, while we agree that making claims of superiority or inferiority using simple comparisons of data from different clinical trials is inappropriate, we believe that referring to and discussing AE rates observed in studies of similar products with acknowledgement of limitations provide a context for the reader to interpret the conveyed information.
